# Novel deep learning radiomics model for preoperative evaluation of hepatocellular carcinoma differentiation based on computed tomography data

**DOI:** 10.1002/ctm2.570

**Published:** 2021-11-06

**Authors:** Yong Ding, Shijian Ruan, Yubizhuo Wang, Jiayuan Shao, Rui Sun, Wuwei Tian, Nan Xiang, Weigang Ge, Xiuming Zhang, Kunkai Su, Jingwen Xia, Qiang Huang, Weihai Liu, Qinxue Sun, Haibo Dong, Mylène C. Q. Farias, Tiannan Guo, Andrey S. Krylov, Wenjie Liang, Wenbo Xiao, Xueli Bai, Tingbo Liang

**Affiliations:** ^1^ Department of Radiology the First Affiliated Hospital Zhejiang University School of Medicine Hangzhou Zhejiang China; ^2^ College of Information Science & Electronic Engineering Zhejiang University Hangzhou Zhejiang China; ^3^ Key Laboratory of Structural Biology of Zhejiang Province School of Life Sciences Westlake University Hangzhou Zhejiang China; ^4^ Center for Infectious Disease Research Westlake Laboratory of Life Sciences and Biomedicine Hangzhou Zhejiang China; ^5^ Institute of Basic Medical Sciences Westlake Institute for Advanced Study Hangzhou Zhejiang China; ^6^ Westlake Omics (Hangzhou) Biotechnology Co., Ltd. Hangzhou Zhejiang China; ^7^ Department of Pathology the First Affiliated Hospital Zhejiang University School of Medicine Hangzhou Zhejiang China; ^8^ State Key Laboratory for Diagnosis and Treatment of Infectious Diseases National Clinical Research Center for Infectious Diseases the First affiliated Hospital School of Medicine Zhejiang University Hangzhou Zhejiang China; ^9^ Department of Radiology The People's Hospital of Beilun District Ningbo China; ^10^ Department of Radiology Ningbo Medical Center Lihuili Hospital Ningbo Zhejiang China; ^11^ Department of Electrical Engineering University of Brasilia Brasília Brazil; ^12^ Laboratory of Mathematical Methods of Image Processing Lomonosov Moscow State University Moscow Russia; ^13^ Department of Hepatobiliary and Pancreatic Surgery The First Affiliated Hospital Zhejiang University School of Medicine Hangzhou Zhejiang China; ^14^ Zhejiang Provincial Key Laboratory of Pancreatic Disease The First Affiliated Hospital Zhejiang University School of Medicine Hangzhou Zhejiang China

Dear Editor,

The evaluation of tumor differentiation is an urgent clinical issue that would facilitate the establishment of individualized therapeutic strategies.^1^, ^2^, ^3^ Our team developed a deep learning radiomics model based on computed tomography (CT) data for preoperative evaluation of hepatocellular carcinoma (HCC) differentiation (low vs. high grade) and preliminarily explored the biological basis of the radiomics model.

We included 1047 patients from the First Affiliated Hospital, College of Medicine, Zhejiang University (Institution 1) and 187 patients from the Ningbo Medical Center Lihuili Hospital (Institution 2). Data from Institution 1 were divided into training and internal validation cohorts by stratified sampling at a 3:1 ratio, while data from Institution 2 constituted the independent test cohort (Figure S1). Patient characteristics are shown in Table 1; there were no significant differences in the distribution of clinical characteristics among the three cohorts.

**TABLE 1:** Patient characteristics of three cohorts

Characteristics	Training cohort	Internal validation cohort	*p* value (training vs. validation)	Independent test cohort	*p* value (training vs. test)
Age (year)	56.56 ± 11.47	56.79 ± 10.62	0.4726	60.32 ± 38.30	0.0867
Sex			0.6997		0.6296
Female	125	42		26	
Male	674	206		161	
Maximum tumor diameter (cm)	4.73 ± 2.92	4.78 ± 2.82	0.2342	4.59 ± 3.12	0.1150
Multiple tumors			0.1575		0.4914
No	678	220		163	
Yes	121	28		24	
Serum AFP level			0.9889		0.3647
Normal	227	71		60	
Abnormal	572	177		127	
Clinical stage			0.9275		0.6359
I/II	692	216		165	
III/IV	107	32		22	
Hepatitis B			0.2578		0.7733
Yes	116	44		25	
No	683	204		162	
Cirrhosis			0.3310		0.0005
Yes	349	99		55	
No	450	149		132	
Symptoms			0.8340		0.0037
Yes	597	183		159	
No	202	65		28	

Abbreviation: AFP, alpha‐fetoprotein.

The radiomics pipeline (Figure 1) mainly involved data acquisition from CT images (Method S1), segmentation of regions of interest, feature extraction (Table S1) and selection, model construction and evaluation and multiomics analysis (Method S2). In total, 707 radiomics features were extracted from CT image data; 614 were filtered out because of low reproducibility or high redundancy, and 25 features with a significant impact on the target were ultimately selected (Table S2). A radiomics signature was established using the random forest (RF) method (Table S3, Figure S2). The AUCs in the training, internal validation and external test cohorts were 0.82, 0.76 and 0.75, respectively (Figure S3). Violin plots of selected features are shown in Figure 4A. The accuracy of the radiomics signature in the training, validation and test cohorts were 0.75, 0.72, and 0.66, respectively; the sensitivity was 0.76, 0.70, and 0.74, respectively; and the specificity was 0.72, 0.75, and 0.54, respectively.

**FIGURE 1:**
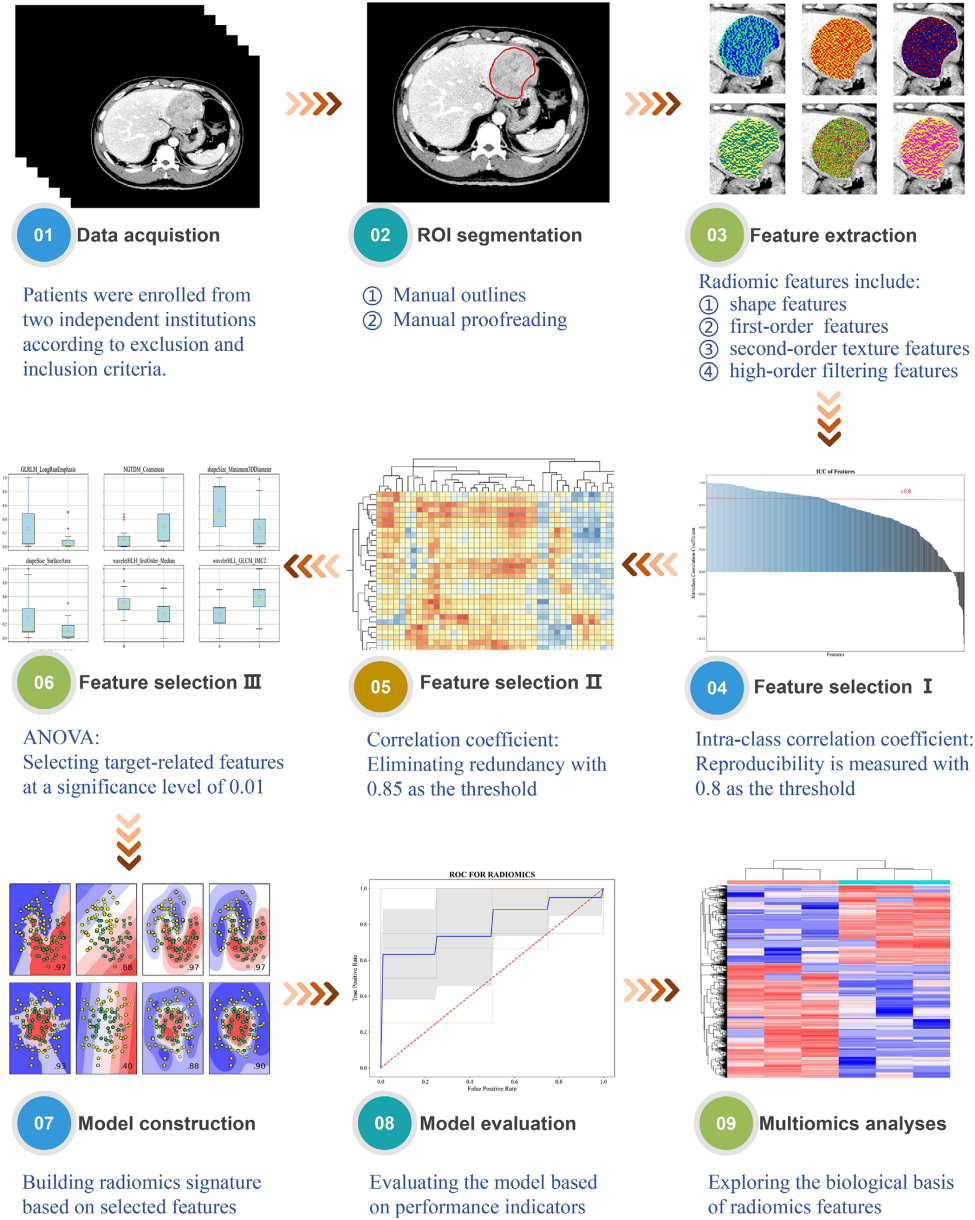
Radiomics module pipeline

The deep learning model in this study was modified from VGG19^4^ (Table S4). A illustration of deep learning model structure is shown in Figure 2. The AUCs of the deep learning model in the training, internal validation and test cohorts were 0.85, 0.81, and 0.75, respectively (Figure S4). The model had an accuracy of 0.77, 0.75, and 0.66, respectively; sensitivity of 0.76, 0.81, and 0.62, respectively; and specificity of 0.66, 0.66, and 0.72, respectively in the three cohorts. In the comparison of the deep learning model with the radiomics signature, *p* values from the DeLong test^5^ were 0.09, 0.17, and 0.62 in the training, validation, and test cohorts, respectively. There were no significant differences between the deep learning model and radiomics signature, although the former had a slightly higher AUC. To see how much value radiomics or deep learning can bring to some risk factors about tumor morphology and size, the features (original_shape2D_Sphericity, original_shape2D_Elongation, original_shape2D_MajorAxisLength) were used to construct a morphological model (Figure S5).

**FIGURE 2:**
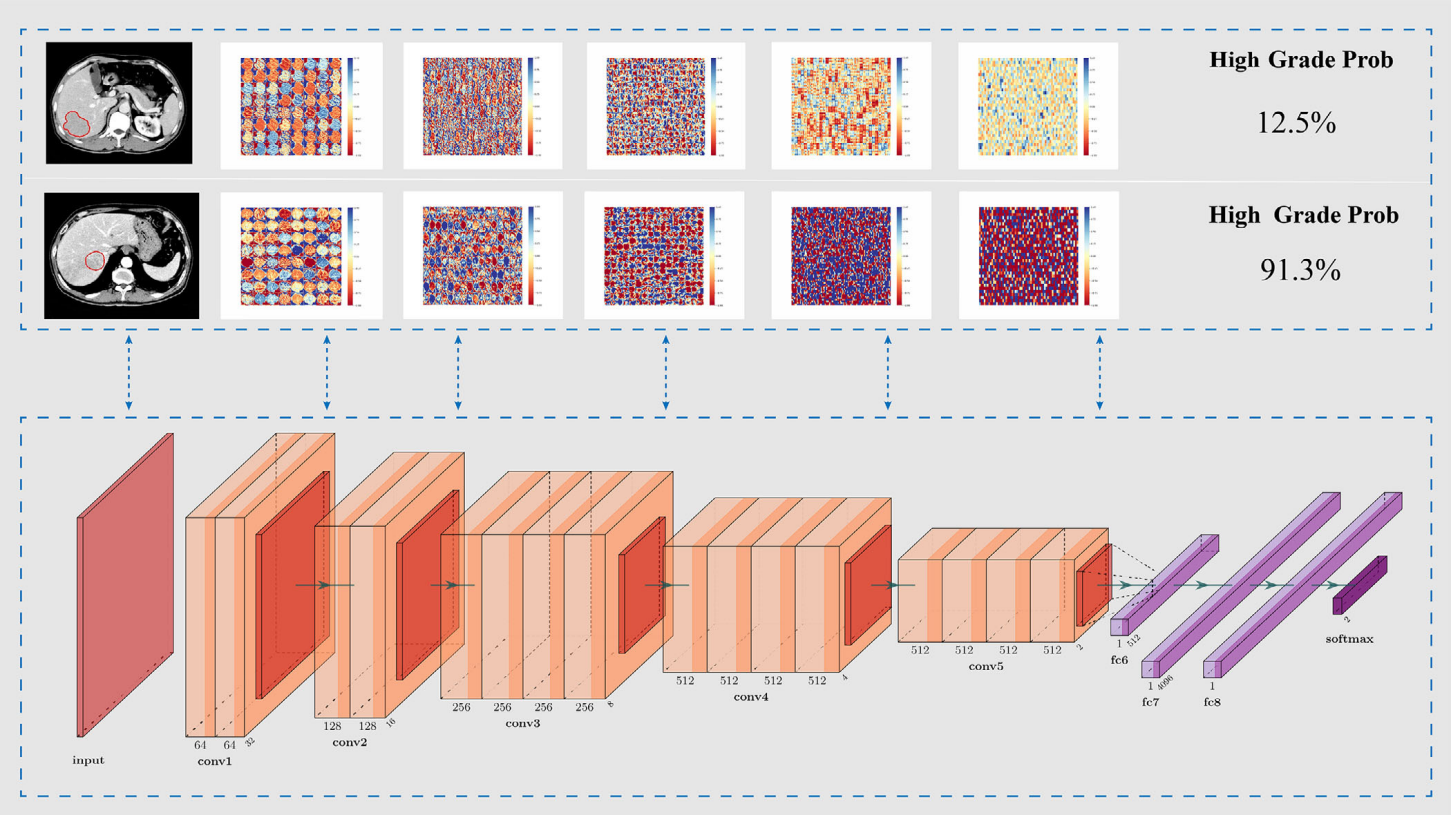
Schematic of deep learning architecture and illustrations of middle layer output splicing of moderately differentiated (upper) and poorly differentiated (lower) cases

Predictions based on clinical characteristics were determined from the clinical model established from RF of clinical characteristics. After visualizing the predicted probabilities of the clinical model, radiomics signature, and deep learning model, we found that the three predictors showed good discriminatory power for groups with different pathologic grades (Figure 3B). The performance of the clinical model is unsatisfactory (Figure S6). Next, the clinical model, radiomics signature, and deep learning model served as the base models for inputting predicted probabilities into the logistic regression model for multi‐model predictions fusion. ROC curves of the fused model applied to the three cohorts are shown in Figure 3C. The results of the DeLong test showed that AUCs of the fused model were significantly improved over those of the base models.

$$\begin{array}{c} FM=-6.81+3.63\times CM+7.51\times RS+3.79\times DL\\ (\mathrm{CM},\;\mathrm{clinical}\;\mathrm{model};\;\mathrm{DL},\;\mathrm{deep}\;\mathrm{learning}\;\mathrm{model};\\ \mathrm{FM},\;\mathrm{fused}\;\mathrm{model};\;\mathrm{RS},\;\mathrm{radiomics}\;\mathrm{signature}) \end{array}$$

**FIGURE 3:**
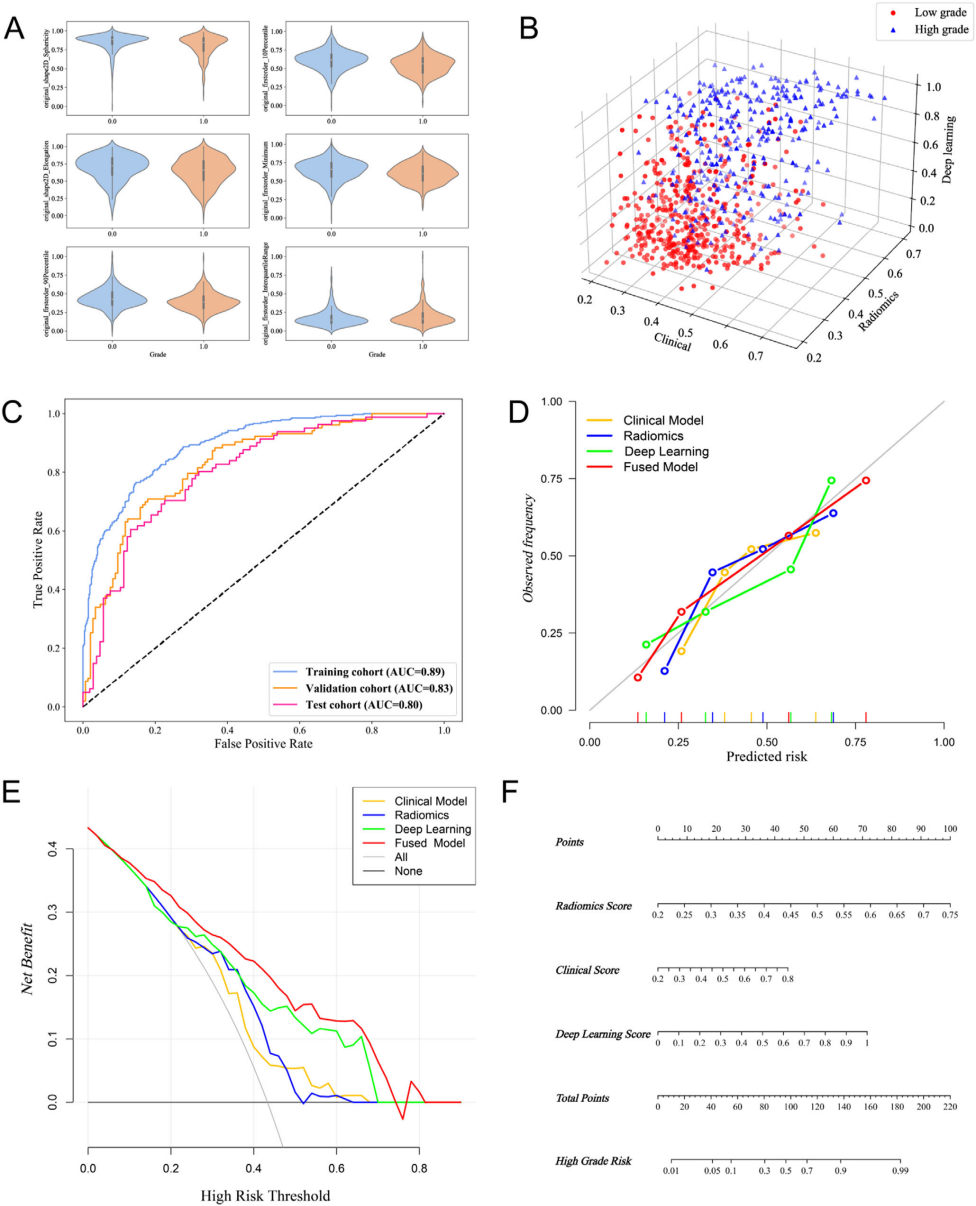
Construction and evaluation of the deep learning radiomics model for preoperative hepatocellular carcinoma (HCC) pathological grading. (A) Violin plots of six selected radiomics features, including ‘original_shape2D_Sphericity’, ‘original_firstorder_10Percentile’, ‘original_shape2D_Elongation’,‘original_firstorder_Minimum’,‘original_glcm_Correlation’ and ‘original_glcm_DifferenceEntropy’. (B) 3D scatterplot of predicted probabilities for the clinical model, radiomics signature, deep learning model and fused model. (C) ROCs and AUCs of the deep learning radiomics model in the three cohorts. (D) Calibration curves of the different models. (E) Decision curves of the different models. (F) Nomogram for preoperative prediction of HCC pathologic grade

Quantitative indices in the comparisons between the clinical model, radiomics signature, deep learning model, and fused model and the results of the DeLong test are summarized in Table 2. The fused model showed the best performance in the training, validation, and test cohorts, with an AUC of 0.89, 0.83, and 0.80, respectively; accuracy of 0.82, 0.77, and 0.73, respectively; sensitivity of 0.85, 0.81, and 0.71, respectively; specificity of 0.76, 0.71, and 0.75, respectively; PPV of 0.84, 0.80, and 0.79, respectively; NPV of 0.78, 0.73, and 0.66, respectively; and F1 score of 0.77, 0.72, and 0.71 respectively. The calibration curves showed that the fused model had better concordance between predicted and actual probabilities than the other models (Figure 3D). Comparison of the decision curves of the four models in the test set indicated that the fused model had greater clinical utility (Figure 3E), and the IDI indicated that the predicted probabilities of the fused model were significantly improved compared to those of the other models (Figure S7). A nomogram for preoperative prediction of HCC pathologic grade was established based on the fused model (Figure 3F).

**TABLE 2:** Comparison of quantitative indices of the clinical model, radiomics signature, deep learning model and fused model applied to the three cohorts

	Training cohort				Internal validation cohort				Independent test cohort			
Methods	CM	RS	DL	FM	CM	RS	DL	FM	CM	RS	DL	FM
AUC	0.7044	0.8223	0.8510	0.8941	0.6264	0.7616	0.8073	0.8301	0.6626	0.7475	0.7513	0.8042
ACC	0.6383	0.7459	0.7735	0.8160	0.6264	0.7177	0.7500	0.7702	0.6150	0.6578	0.6631	0.7273
SENS	0.6157	0.7622	0.8535	0.8535	0.5724	0.6965	0.8137	0.8137	0.6698	0.7453	0.6226	0.7075
SPEC	0.6707	0.7225	0.6585	0.7622	0.5825	0.7476	0.6601	0.7087	0.5432	0.5432	0.7160	0.7531
PPV	0.7286	0.7978	0.7821	0.8375	0.6587	0.7953	0.7712	0.7973	0.6574	0.6810	0.7416	0.7895
NPV	0.5486	0.6790	0.7578	0.7837	0.4918	0.6363	0.7157	0.7300	0.5570	0.6197	0.5918	0.6630
F1 score	0.6036	0.7001	0.7047	0.7728	0.5333	0.6875	0.6868	0.7192	0.5499	0.5789	0.6480	0.7052
Significance level of DeLong test for models compared with FM												

	CM	RS	DL
Training cohort	<0.0001	<0.0001	<0.0001
Internal validation cohort	<0.0001	0.0035	0.1083
Independent test cohort	0.0005	0.0295	0.0132

Abbreviations: AUC, area under curve; ACC, accuracy; CM, clinical model; DL, deep learning model; FM, fused model; NPV, negative predictive value; PPV, positive predictive value; RS, radiomics signature; SENS: sensitivity; SPEC, specificity.

A total of 69 patients with CT data were included in the multiomics analysis. After data preprocessing, 19723 genomics, 42807 transcriptomics, and 3658 proteomics variables with differential expression between high‐ and low‐grade HCC (valid data > 80%) were extracted. Pearson's correlation coefficients between radiomics features and multiomics variables are shown as correlation heat maps (Figure 4A). The selected radiomics features reconstructed 65.54%, 64.65%, and 72.69% of the differentially expressed genes, transcripts, and proteins (Figure 4B). The coverage of each type of ‐omics was 60% with just 15 radiomics features. The radiomics‐related multiomics variables showed significant differences between the different pathologic grades (high vs. low grade) (Figure 4C).

**FIGURE 4:**
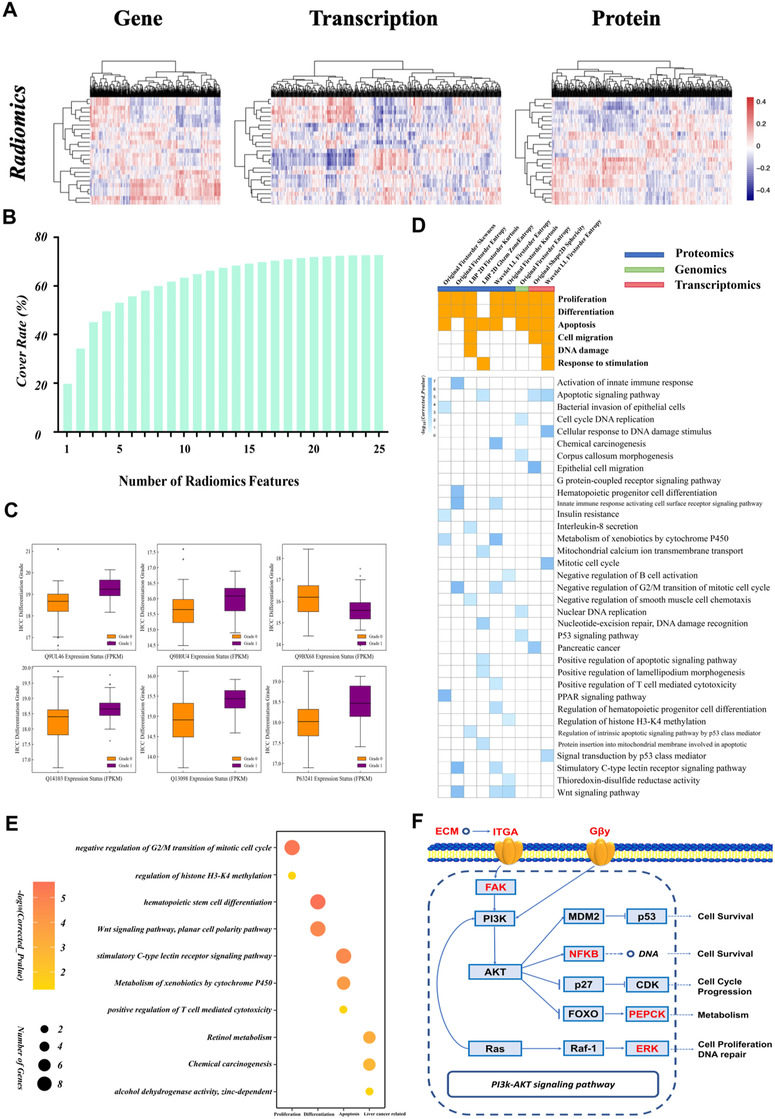
Results of the multiomics analysis. (A) Heat map of correlations between radiomics features and multiomics (genomics, transcriptomics and proteomics) variables. (B) Histogram of the relationship between the number of radiomics features and coverage of biological variables identified from multiomics analyses. (C) Box plots of specific proteins related to selected radiomics features showing significant differences in expression between high‐ and low‐grade groups. (D) Matrices of cancer‐related biological processes covered by radiomics features at specific ‐omics levels (upper); and details of GO terms and pathways (lower). (E) Bubble chart of 10 important GO terms and pathways correlated with wavelet_LL_firstorder_entropy used to establish the radiomics signature. The biological process of each GO term or pathway is shown on the x‐axis. (F) Key genes (red) in the phosphatidylinositol 3‐kinase (PI3K)/protein kinase B (AKT) signaling pathway were reconstructed with original_shape2D_sphericity, which was used to establish the radiomics signature

The results of the gene enrichment analysis of 25 radiomics features are summarized in Figure 4D. In the enrichment result for wavelet_LL_first‐order_entropy, 21 GO terms and pathways were identified that are potentially related to HCC development. For example, wavelet_LL_first‐order_entropy was associated with abnormal alcohol dehydrogenase activity, which leads to abnormal development and cell apoptosis. Key genes associated with original_shape2D_sphericity were related to the phosphatidylinositol 3‐kinase (PI3K)/protein kinase B (AKT) signaling pathway (Figure 4F), which is involved in apoptosis, cancer cell proliferation, DNA repair, and cancer differentiation, among other biological processes.

In conclusion, we established a deep learning radiomics model that can be used for preoperative pathological grading of HCC and served as a noninvasive prediction tool to guide clinical decision‐making.

## CONFLICT OF INTEREST

The authors declare that they have no competing interests.

## Supporting information

Supporting information
