# Supplementary material for: Novel deep learning radiomics model for preoperative evaluation of hepatocellular carcinoma differentiation based on computed tomography data
Source: Clin Transl Med. 2021 Nov 6;11(11):e570. doi: 10.1002/ctm2.570 (PMC8571950; doi:10.1002/ctm2.570)
Supplement: Supplementary file 1 — Supporting information [file CTM2-11-e570-s001.docx]

Appendices

[Method S1. Image acquisition 2](#_Toc26116)

[Method S2. Multiomics analysis 2](#_Toc863)

*[(1) Transcriptome sequencing](#_Toc21978)* [2](#_Toc21978)

*[(2) Proteomic analysis](#_Toc1081)* [3](#_Toc1081)

*[(3) Multiomics analysis](#_Toc22363)* [3](#_Toc22363)

[References 5](#_Toc5159)

[Figure S1. Flowchart of the study protocol 6](#_Toc2142)

[Figure S2. Importance of clinical characteristics in clinical model based on random forest. 7](#_Toc15688)

[Figure S3. ROC curves and AUCs for the radiomics signature. 8](#_Toc10204)

[Figure S4. ROC curves and AUCs for the deep learning model. 9](#_Toc5127)

[Figure S5. ROC curves and AUCs for the clinical model. 10](#_Toc18237)

[Figure S6. Graphical depiction of integrated discrimination improvement (IDI). 11](#_Toc5157)

[Table S1. Quantitative radiomics features. 12](#_Toc20675)

[Table S2. Analysis of Variance (ANOVA) for radiomics features between low grade group and high grade group. 13](#_Toc27675)

[Table S3. The important parameters for model construction based on random forest. 16](#_Toc13551)

[Table S4. The structure of deep learning network. 17](#_Toc7818)

Method S1. Image acquisition

Contrast-enhanced CT scans were performed for all patients with HCC at the two participating institutions. CT scans at Institution 1 were performed on a 16-slice (Aquilion; Toshiba Medical Systems, Tokyo, Japan) or 256-slice (Brilliance iCT; Philips Healthcare, Cleveland, OH, USA) CT scanner. Scans at Institution 2 were performed on a 16-slice Brilliance scanner (Philips Medical Systems, Andover, MA, USA). The image acquisition parameters were as follows: tube voltage=100–120 kVp, tube current=200–320 mAs), matrix=512×512, and slice thickness=0.625–5 mm. The nonionic contrast agents included iohexol (Kang Chen Pharmaceutical Group, Guangzhou, China and Yangtze River Pharmaceutical Group, Taizhou, China) and ioversol (Hengrui Medicine, Lianyungang, China). Arterial phase (25–38s), portal venous phase (55–85 s), and delay phase (120–180 s) CT scans were performed after administration of the contrast agent (1.5 ml/kg) by bolus injection (3.0 ml/s) into the forearm vein. Only venous phase CT images of HCC were used for delineation and analysis in this study.

Method S2. Multiomics analysis*[1-6]*

1. *Transcriptome sequencing*

A. RNA quantification and qualification

RNA degradation and contamination was monitored on 1% agarose gels; RNA purity was checked using the NanoPhotometer^®^ spectrophotometer (IMPLEN, CA,USA); RNA integrity was assessed using the RNA Nano 6000 Assay Kit of the Bioanalyzer 2100 system (Agilent Technologies, CA, USA).

1. Library preparation for Transcriptome sequencing

A total amount of 1 µg RNA per sample was used as input material for the RNA sample preparations. Sequencing libraries were generated using NEBNext^®^ UltraTM RNA Library Prep Kit for Illumina^®^ (NEB, USA) following manufacturer’s recommendations and index codes were added to attribute sequences to each sample. Briefly, mRNA was purified from total RNA using poly-T oligo-attached magnetic beads. Fragmentation was carried out using divalent cations under elevated temperature in NEBNext First Strand Synthesis Reaction Buffer (5X). First strand cDNA was synthesized using random hexamer primer and M-MuLV Reverse Transcriptase (RNase H^-^). Second strand cDNA synthesis was subsequently performed using DNA Polymerase I and RNase H. Remaining overhangs were converted into blunt ends via exonuclease/polymerase activities. After adenylation of 3’ ends of DNA fragments, NEBNext Adaptor with hairpin loop structure were ligated to prepare for hybridization. In order to select cDNA fragments of preferentially 250~300 bp in length, the library fragments were purified with AMPure XP system (Beckman Coulter, Beverly, USA). Then 3 µl USER Enzyme (NEB, USA) was used with size-selected, adaptor-ligated cDNA at 37℃ for 15 m in followed by 5 min at 95℃ before PCR. Then PCR was performed with Phusion High -Fidelity DNA polymerase, Universal PCR primers and Index (X) Primer. At last, PCR products were purified (AMPure XP system) and library quality was assessed on the Agilent Bioanalyzer 2100 system.

(3) Clustering and sequencing

The clustering of the index-coded samples was performed on a cBot Cluster Generation System using TruSeq PE Cluster Kit v3-cBot-HS (Illumia) according to the manufacturer’s instructions. After cluster generation, the library preparations were sequenced on an Illumina Novaseq platform and 150 bp paired-end reads were generated.

1. *Proteomic analysis*

Each fresh frozen tissue sample was denatured with 6 M urea (Sigma-Aldrich, Germany) and 2 M thiourea (Sigma-Aldrich, Germany) using pressure-cycling technology (PCT) assisted method and were digested into peptides with trypsin (Hualishi, Beijing, China) and Lys-C (Hualishi, Beijing, China) [1-3]. 500 ng peptides were separated with UltimateTM 3000 nano LC (Dionex LC-Packings, Amsterdam, The Netherlands) and then analyzed with a Q Exactive HF mass spectrometry by data-independent acquisition (DIA) mode (Thermo Fisher Scientific™, San Jose, USA) as described previously [1]. The raw files were analyzed with SpectronautTM (version: 14.5.200813.47784) using the optimized DPHL spectrum library [4]. False discovery rate was controlled below 1% at both protein and peptide level. Two-sided unpaired Welch’s t test was performed for each pair of comparing groups. Adjusted p-values were calculated using Benjamini & Hochberg correction. Protein regulations with adjusted p-value < 0.05 and log2(fold change) higher than 1.2 were considered as significant. R package Mfuzz was used for clustering for the four groups [5]. Gene ontology (GO) and Kyoto encyclopedia of genes and genomes (KEGG) pathways were enriched on the metascape web [6].

1. *Multiomics analysis*

Tumor tissue samples from 69 HCC patients obtained by surgical excision were immediately frozen in liquid nitrogen and transferred to a freezer at −80°C. The samples were subjected to transcriptome sequencing and proteome analysis. Multiomics (genomics, transcriptomics, proteomics) variables that were differentially expressed between high- and low-grade HCC were extracted by preprocessing. The coverage of multiomics variables was calculated. We performed a Pearson correlation analysis to evaluate the relationship between radiomics features and multiomics data; those showing a high correlation were selected for gene enrichment analysis. Gene Ontology (GO) terms and pathways related to specific radiomics features from different -omics datasets were classified into 6 categories (proliferation, differentiation, apoptosis, cell migration, DNA damage, and response to stimulation) according to the associated biological process. The biological significance of radiomics features was further analyzed by investigating the GO terms and pathways related to the proteomics data. Key genes in key pathways related to cancer differentiation are presented in a pathway map.

**References**

[1] Gao HH, Zhang FF, Liang S, Zhang QS, Lyu MG, Qian LJ, et al. Accelerated Lysis and Proteolytic Digestion of Biopsy-Level Fresh-Frozen and FFPE Tissue Samples Using Pressure Cycling Technology. J Proteome Res 2020;19(5):1982-90.

[2] Guo TN, Kouvonen P, Koh CC, Gillet LC, Wolski WE, Rost HL, et al. Rapid mass spectrometric conversion of tissue biopsy samples into permanent quantitative digital proteome maps. Nat Med 2015;21(4):407-413.

[3] Zhu Y, Weiss T, Zhang QS, Sun R, Wang B, Yi X, et al. High-throughput proteomic analysis of FFPE tissue samples facilitates tumor stratification. Mol Oncol 2019;13(11):2305-28.

[4] Zhu TS, Zhu Y, Xuan Y, Gao HH, Cai X, Piersma SR, et al. DPHL: A DIA Pan-human Protein Mass Spectrometry Library for Robust Biomarker Discovery. Genom Proteom Bioinf 2020;18(2):104-19.

[5] Kumar L, M EF. Mfuzz: a software package for soft clustering of microarray data. Bioinformation 2007;2(1):5-7.

[6] Zhou Y, Zhou B, Pache L, Chang M, Khodabakhshi AH, Tanaseichuk O, et al. Metascape provides a biologist-oriented resource for the analysis of systems-level datasets. Nat Commun 2019;10(1):1523

# Figure S1. Flowchart of the study protocol

#
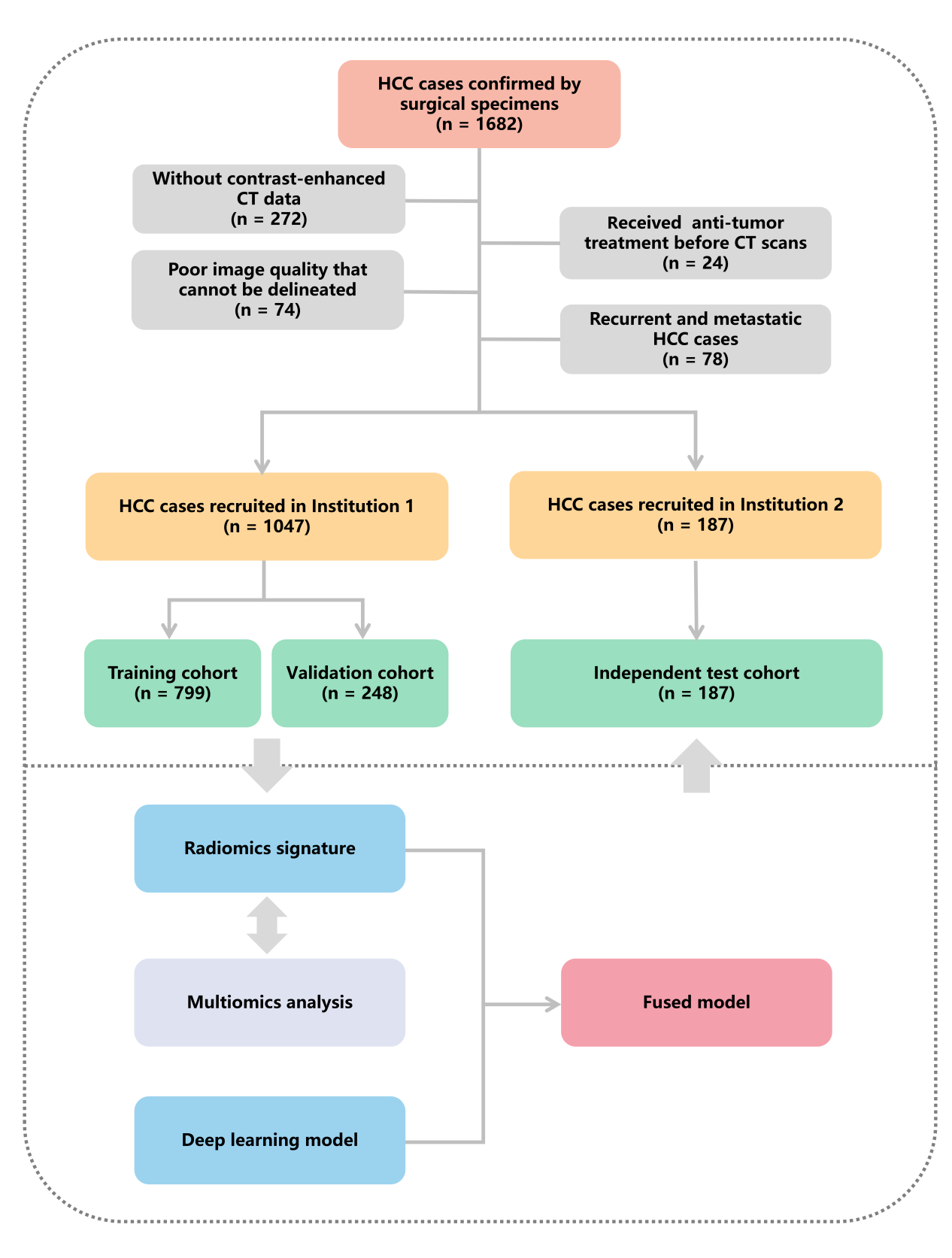


# Figure S2. Importance of clinical characteristics in clinical model based on random forest.


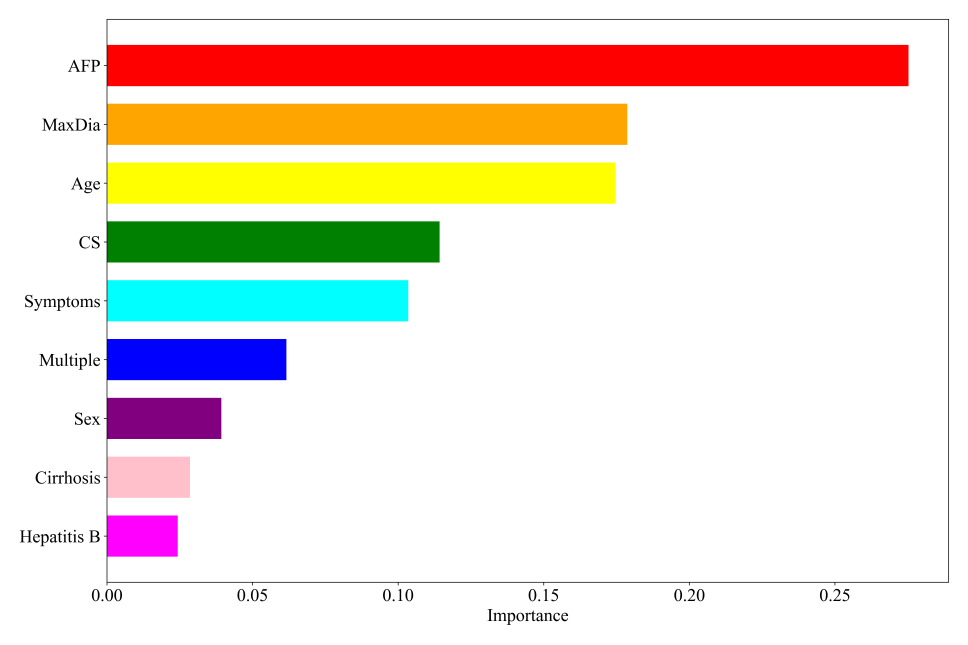


Multiple, multiple tumors; Maxdia, maximum tumor diameter; AFP, serum alpha-fetoprotein level; CS, clinical stage.

# Figure S3. ROC curves and AUCs for the radiomics signature.


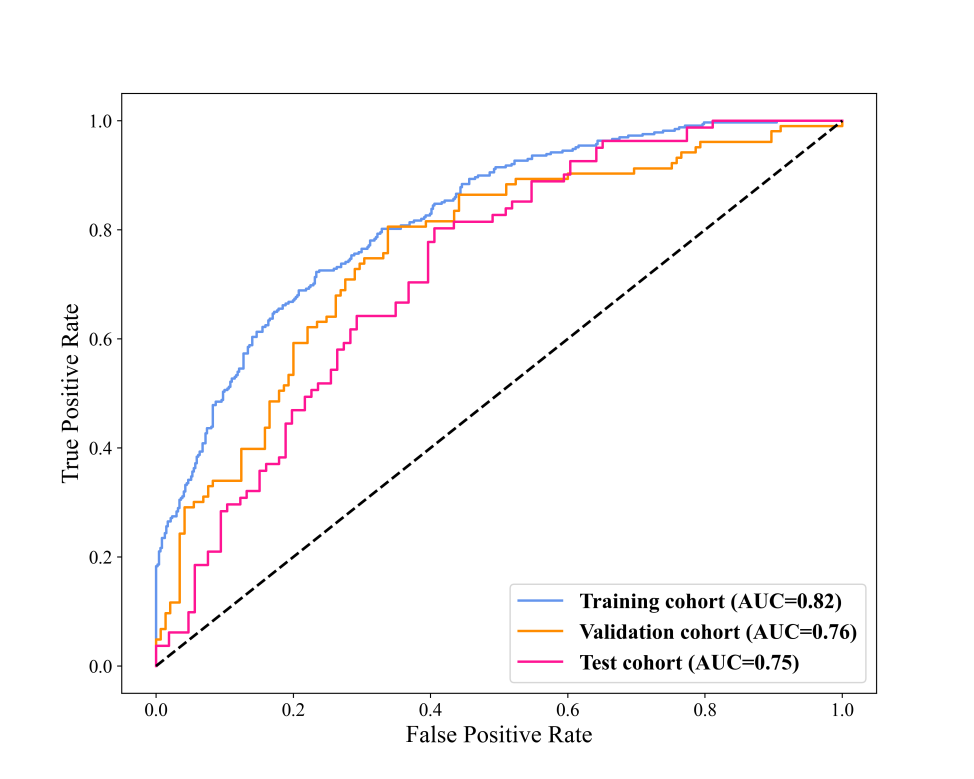


# Figure S4. ROC curves and AUCs for the deep learning model.


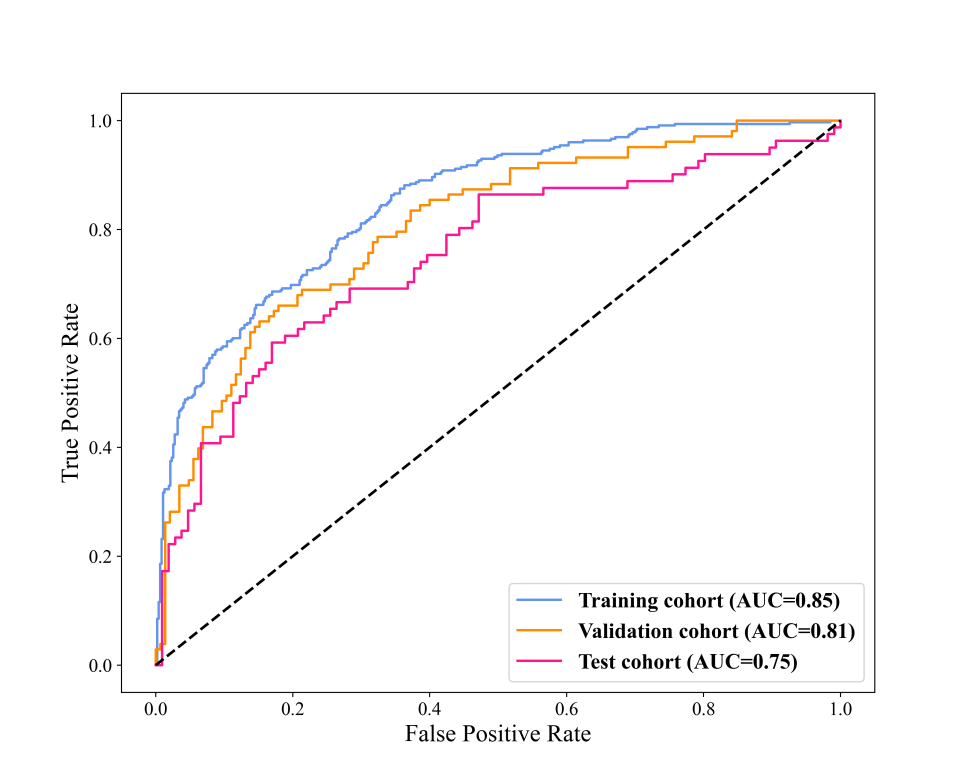


# Figure S5. ROC curves and AUCs for the morphological model.

#
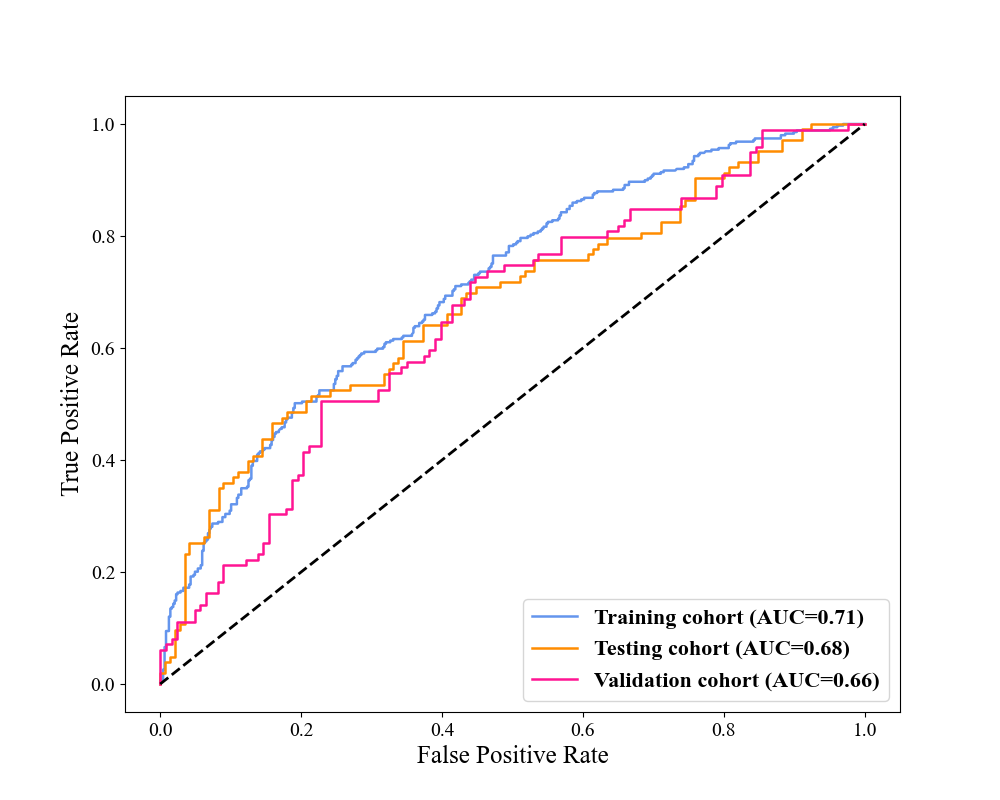


# Figure S6. ROC curves and AUCs for the clinical model.


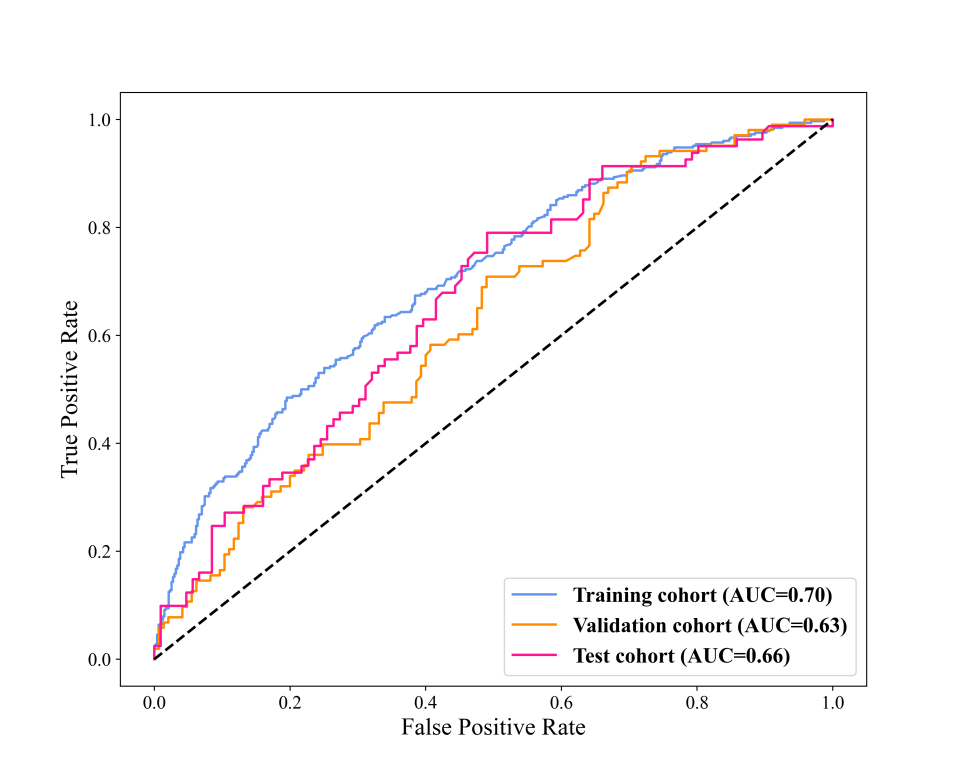


# Figure S7. Graphical depiction of integrated discrimination improvement (IDI).


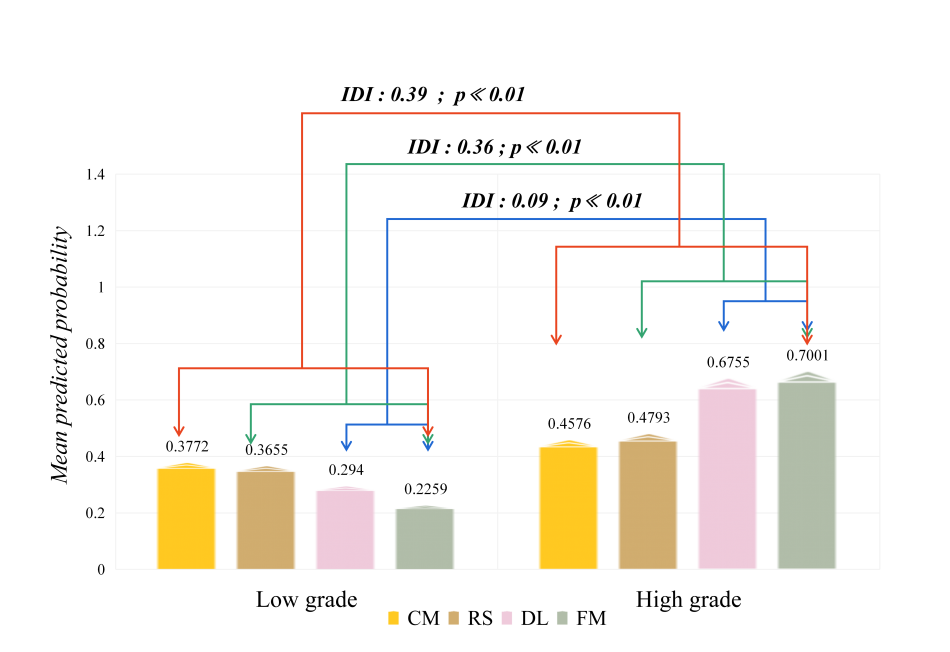


The predicted probabilities of the fused model were significantly improved over other models (clinical model, radiomics signature and deep learning model). CM, clinical model; RS, radiomics signature; DL, deep learning model; FM, fused model.

|  |
| --- |

# Table S1. Quantitative radiomics features.

| **Type** | **Details** |
| --- | --- |
| **Shape features** | Mesh Surface, Pixel Surface, Perimeter, Perimeter to Surface ratio, Sphericity, Spherical Disproportion, Maximum 2D diameter, Major Axis Length, Minor Axis Length, Elongation; define the shape of the tumor. |
| **First-order features** | Energy, Total Energy, Entropy, Minimum, 10th percentile, 90th percentile, Maximum, Mean, Median, Interquartile Range, Range, Mean Absolute Deviation (MAD), Robust Mean Absolute Deviation (rMAD), Root Mean Squared (RMS), Standard Deviation, Skewness, Kurtosis, Variance, Uniformity; represent the distribution of pixels without considering spatial relationship. |
| **Second-order texture features** | GLCM, GLSZM, GLRLM, NGTDM, GLDM; extracting features after coding the spatial relationship between pixels. |
| **High-order filtering features** | **Wavelet**: wavelet filtering, yields 4 decompositions per level by applying a high or low pass filter in different dimensions, reflects the structural characteristics at different image frequencies (LL-, LH-, HL-, HH-). |
|  | **LBP**: Local Binary Pattern can be used to get the local texture characteristics of an image, which has significant advantages of invariance to grayscale and rotation. |

**Note.** GLCM, gray-level co-occurrence matrix; GLSZM, gray-level size zone matrix; GLRLM, gray-level run length matrix; NGTDM, neighboring gray tone difference matrix; GLDM, gray-level dependence matrix; LBP, local binary pattern.

# Table S2. Analysis of Variance (ANOVA) for radiomics features between low grade group and high grade group.

| **Features** | **p value** | **Score** |
| --- | --- | --- |
| original_shape2D_Sphericity | 1.40E-11 | 46.56 |
| original_firstorder_10Percentile | 3.35E-09 | 35.49 |
| original_shape2D_Elongation | 1.14E-06 | 23.92 |
| original_firstorder_Minimum | 3.00E-05 | 17.55 |
| original_firstorder_90Percentile | 1.50E-04 | 14.46 |
| original_firstorder_InterquartileRange | 5.46E-04 | 12.02 |
| original_glcm_Correlation | 8.80E-04 | 11.12 |
| original_firstorder_TotalEnergy | 1.16E-03 | 10.61 |
| original_shape2D_MajorAxisLength | 1.32E-03 | 10.36 |
| original_firstorder_Skewness | 1.55E-03 | 10.06 |
| wavelet-LH_firstorder_Mean | 1.63E-03 | 9.97 |
| original_glcm_Autocorrelation | 2.77E-03 | 8.99 |
| wavelet-LL_ngtdm_Complexity | 5.82E-03 | 7.63 |
| wavelet-HL_firstorder_Median | 6.26E-03 | 7.50 |
| original_glcm_DifferenceEntropy | 7.87E-03 | 7.09 |
| original_firstorder_Range | 1.36E-02 | 6.10 |
| original_firstorder_Entropy | 1.42E-02 | 6.03 |
| lbp-2D_firstorder_Kurtosis | 1.76E-02 | 5.65 |
| original_glcm_SumEntropy | 1.97E-02 | 5.45 |
| original_glcm_Contrast | 2.05E-02 | 5.39 |
| lbp-2D_glszm_ZoneEntropy | 3.33E-02 | 4.54 |
| wavelet-LL_firstorder_Entropy | 4.03E-02 | 4.22 |
| wavelet-LH_firstorder_Median | 4.66E-02 | 3.97 |
| original_firstorder_Kurtosis | 4.79E-02 | 3.92 |
| wavelet-HH_firstorder_Median | 4.82E-02 | 3.91 |
| lbp-2D_firstorder_InterquartileRange | 5.62E-02 | 3.65 |
| wavelet-HL_glszm_LargeAreaLowGrayLevelEmphasis | 5.77E-02 | 3.61 |
| wavelet-HL_firstorder_Mean | 6.07E-02 | 3.53 |
| wavelet-HH_firstorder_Maximum | 6.55E-02 | 3.40 |
| wavelet-HH_firstorder_10Percentile | 7.66E-02 | 3.14 |
| original_ngtdm_Complexity | 8.87E-02 | 2.90 |
| lbp-2D_glcm_InverseVariance | 9.10E-02 | 2.86 |
| lbp-2D_glszm_SizeZoneNonUniformityNormalized | 1.04E-01 | 2.64 |
| wavelet-HL_glrlm_LongRunLowGrayLevelEmphasis | 1.21E-01 | 2.40 |
| wavelet-HH_glcm_ClusterShade | 1.32E-01 | 2.28 |
| wavelet-LH_glcm_ClusterShade | 1.44E-01 | 2.14 |
| wavelet-LH_ngtdm_Complexity | 1.75E-01 | 1.84 |
| wavelet-LH_firstorder_Minimum | 1.93E-01 | 1.70 |
| wavelet-HL_glcm_Autocorrelation | 1.99E-01 | 1.65 |
| original_glcm_ClusterShade | 2.49E-01 | 1.33 |
| wavelet-LH_firstorder_Kurtosis | 2.79E-01 | 1.18 |
| lbp-2D_glcm_DifferenceEntropy | 3.02E-01 | 1.07 |
| wavelet-HH_firstorder_Entropy | 3.08E-01 | 1.04 |
| lbp-2D_firstorder_Median | 3.15E-01 | 1.01 |
| wavelet-HL_firstorder_Kurtosis | 3.16E-01 | 1.01 |
| lbp-2D_firstorder_Entropy | 3.17E-01 | 1.00 |
| lbp-2D_glszm_SmallAreaHighGrayLevelEmphasis | 3.31E-01 | 0.95 |
| wavelet-LH_firstorder_Maximum | 3.38E-01 | 0.92 |
| wavelet-HL_glcm_JointEnergy | 3.44E-01 | 0.90 |
| wavelet-HL_glcm_Correlation | 3.54E-01 | 0.86 |
| original_ngtdm_Contrast | 3.69E-01 | 0.81 |
| original_firstorder_Maximum | 3.76E-01 | 0.78 |
| original_glcm_JointEnergy | 3.80E-01 | 0.77 |
| lbp-2D_firstorder_90Percentile | 3.95E-01 | 0.72 |
| original_shape2D_PerimeterSurfaceRatio | 4.34E-01 | 0.61 |
| wavelet-LH_glrlm_LongRunLowGrayLevelEmphasis | 4.44E-01 | 0.59 |
| wavelet-HH_glcm_Autocorrelation | 4.51E-01 | 0.57 |
| wavelet-HL_firstorder_Minimum | 4.71E-01 | 0.52 |
| lbp-2D_glcm_Idm | 4.72E-01 | 0.52 |
| wavelet-LL_glrlm_LongRunLowGrayLevelEmphasis | 4.81E-01 | 0.50 |
| wavelet-HL_firstorder_Entropy | 4.86E-01 | 0.49 |
| wavelet-LH_firstorder_Entropy | 5.03E-01 | 0.45 |
| wavelet-HL_firstorder_Maximum | 5.24E-01 | 0.41 |
| wavelet-LH_glszm_ZoneEntropy | 5.25E-01 | 0.40 |
| wavelet-HH_glcm_Correlation | 5.50E-01 | 0.36 |
| wavelet-HL_firstorder_10Percentile | 5.54E-01 | 0.35 |
| wavelet-HH_ngtdm_Complexity | 5.56E-01 | 0.35 |
| wavelet-HL_glcm_ClusterShade | 6.01E-01 | 0.27 |
| wavelet-LH_firstorder_Skewness | 6.20E-01 | 0.25 |
| wavelet-LH_firstorder_10Percentile | 6.23E-01 | 0.24 |
| wavelet-LH_glcm_Autocorrelation | 6.43E-01 | 0.22 |
| wavelet-HL_glszm_ZoneEntropy | 6.74E-01 | 0.18 |
| wavelet-HH_glszm_LargeAreaHighGrayLevelEmphasis | 6.91E-01 | 0.16 |
| wavelet-HL_glcm_ClusterProminence | 7.09E-01 | 0.14 |
| lbp-2D_glszm_HighGrayLevelZoneEmphasis | 7.47E-01 | 0.10 |
| wavelet-HH_firstorder_Skewness | 7.54E-01 | 0.10 |
| wavelet-HL_glszm_LargeAreaHighGrayLevelEmphasis | 7.76E-01 | 0.08 |
| original_glcm_Imc2 | 7.85E-01 | 0.07 |
| wavelet-HL_firstorder_Skewness | 7.86E-01 | 0.07 |
| wavelet-HL_ngtdm_Complexity | 7.87E-01 | 0.07 |
| lbp-2D_glcm_Correlation | 7.94E-01 | 0.07 |
| wavelet-LL_glszm_LargeAreaEmphasis | 8.02E-01 | 0.06 |
| wavelet-LH_glcm_Correlation | 8.08E-01 | 0.06 |
| original_glcm_ClusterProminence | 8.11E-01 | 0.06 |
| lbp-2D_firstorder_Mean | 8.67E-01 | 0.03 |
| wavelet-HH_glszm_ZoneEntropy | 8.81E-01 | 0.02 |
| wavelet-HH_glrlm_LongRunLowGrayLevelEmphasis | 9.03E-01 | 0.01 |
| lbp-2D_firstorder_10Percentile | 9.12E-01 | 0.01 |
| lbp-2D_gldm_DependenceVariance | 9.20E-01 | 0.01 |
| original_glrlm_LongRunLowGrayLevelEmphasis | 9.55E-01 | 0.00 |
| wavelet-LH_glcm_ClusterProminence | 9.61E-01 | 0.00 |
| lbp-2D_glszm_LargeAreaEmphasis | 9.65E-01 | 0.00 |
| wavelet-HH_glcm_ClusterProminence | 9.74E-01 | 0.00 |

# Table S3. The important parameters for model construction based on random forest.

| **Model** | **Important parameters** |
| --- | --- |
| Clinical model | n_estimators=40, criterion='gini', max_depth=6, min_samples_leaf=10 |
| Radiomics signature | n_estimators=60, criterion='gini', max_depth=4, min_samples_leaf=10 |

# Table S4. The structure of deep learning network.

| Layer name | Output size | **Layer design (size, filter)** | activation |
| --- | --- | --- | --- |
| block1_conv1 | 32×32 | 3×3 conv, 64 | ReLU |
| block1_conv2 | 32×32 | 3×3 conv, 64 | ReLU |
| block1_pool | 16×16 | Max 2×2, stride 2 ("same") |  |
| block2_conv1 | 16×16 | 3×3 conv, 128 | ReLU |
| block2_conv2 | 16×16 | 3×3 conv, 128 | ReLU |
| block2_pool | 8×8 | Max 2×2, stride 2 ("same") |  |
| block3_conv1 | 8×8 | 3×3 conv, 256 | ReLU |
| block3_conv2 | 8×8 | 3×3 conv, 256 | ReLU |
| block3_conv3 | 8×8 | 3×3 conv, 256 | ReLU |
| block3_conv4 | 8×8 | 3×3 conv, 256 | ReLU |
| block3_pool | 4×4 | Max 2×2, stride 2 ("same") |  |
| block4_conv1 | 4×4 | 3×3 conv, 512 | ReLU |
| block4_conv2 | 4×4 | 3×3 conv, 512 | ReLU |
| block4_conv3 | 4×4 | 3×3 conv, 512 | ReLU |
| block4_conv4 | 4×4 | 3×3 conv, 512 | ReLU |
| block4_pool | 2×2 | Max 2×2, stride 2 ("same") |  |
| block5_conv1 | 2×2 | 3×3 conv, 512 | ReLU |
| block5_conv2 | 2×2 | 3×3 conv, 512 | ReLU |
| block5_conv3 | 2×2 | 3×3 conv, 512 | ReLU |
| block5_conv4 | 2×2 | 3×3 conv, 512 | ReLU |
| flatten | 2048 | 2×2×512 |  |
| fc1 | 4096 | 2048×4096 | ReLU |
| Dropout 0.5 | | | |
| fc2 | 4096 | 4096×4096 | ReLU |
| Dropout 0.5 | | | |
| predictions | 2 | 4096×2 | softmax |

|  |
| --- |
|  |

**Note.** conv, convolution; fc, full connection layer; ReLU, rectified linear unit.
